# Hedgehog Signaling Antagonist GDC-0449 (Vismodegib) Inhibits Pancreatic Cancer Stem Cell Characteristics: Molecular Mechanisms

**DOI:** 10.1371/journal.pone.0027306

**Published:** 2011-11-08

**Authors:** Brahma N. Singh, Junsheng Fu, Rakesh K. Srivastava, Sharmila Shankar

**Affiliations:** 1 Department of Pharmacology, Toxicology and Therapeutics, and Medicine, The University of Kansas Cancer Center, The University of Kansas Medical Center, Kansas City, Kansas, United States of America; 2 Department of Pathology and Laboratory Medicine, The University of Kansas Cancer Center, The University of Kansas Medical Center, Kansas City, Kansas, United States of America; Wayne State University, United States of America

## Abstract

**Background:**

Recent evidence from *in vitro* and *in vivo* studies has demonstrated that aberrant reactivation of the Sonic Hedgehog (SHH) signaling pathway regulates genes that promote cellular proliferation in various human cancer stem cells (CSCs). Therefore, the chemotherapeutic agents that inhibit activation of Gli transcription factors have emerged as promising novel therapeutic drugs for pancreatic cancer. GDC-0449 (Vismodegib), orally administrable molecule belonging to the 2-arylpyridine class, inhibits SHH signaling pathway by blocking the activities of Smoothened. The objectives of this study were to examine the molecular mechanisms by which GDC-0449 regulates human pancreatic CSC characteristics *in vitro*.

**Methodology/Principal Findings:**

GDC-0499 inhibited cell viability and induced apoptosis in three pancreatic cancer cell lines and pancreatic CSCs. This inhibitor also suppressed cell viability, Gli-DNA binding and transcriptional activities, and induced apoptosis through caspase-3 activation and PARP cleavage in pancreatic CSCs. GDC-0449-induced apoptosis in CSCs showed increased Fas expression and decreased expression of PDGFRα. Furthermore, Bcl-2 was down-regulated whereas TRAIL-R1/DR4 and TRAIL-R2/DR5 expression was increased following the treatment of CSCs with GDC-0449. Suppression of both Gli1 plus Gli2 by shRNA mimicked the changes in cell viability, spheroid formation, apoptosis and gene expression observed in GDC-0449-treated pancreatic CSCs. Thus, activated Gli genes repress DRs and Fas expressions, up-regulate the expressions of Bcl-2 and PDGFRα and facilitate cell survival.

**Conclusions/Significance:**

These data suggest that GDC-0499 can be used for the management of pancreatic cancer by targeting pancreatic CSCs.

## Introduction

Pancreatic cancer (PC) is a highly lethal malignancy characterized by late diagnosis and treatment resistance. PC is the fourth leading cause of cancer in the United States with a 5-year survival less than 5%. Surgical resection is the only potentially curative therapeutic option for PC; however, due to the lack of early symptoms, the vast majority of patients present with metastatic disease, rendering their malignancy inoperable [1], [2]. The current standard-of-care therapy, gemcitabine, extends patient survival by only a few weeks [3]. The identification of new molecular targets for PC to overcome the dismal prognosis is therefore necessary. Recurrent genetic alterations in defined genes in association with perturbations of developmental cell signaling pathways have been associated with PC development and progression. Recent evidence from *in vitro* and *in vivo* studies suggests that the Sonic Hedgehog (SHH) signaling pathway [4] is aberrantly reactivated and recognized as one of the mediators in the majority of PCs; therefore, SHH blockade has the potential to prevent disease progression and metastatic spread [5].

SHH signaling is initiated by the binding of short-acting polypeptide ligand namely Shh (Sonic Hedgehog, Indian Hedgehog or Desert Hedgehog) to its receptor, Patched which thereby, diminishes the inhibitory effects of Patched on Smoothened [6]. Smoothened is then localized into the primary cilium of the cell, an organelle playing a critical role in SHH signaling [7]. There, Smoothened activates an intracellular cascade that results in activation and nuclear translocation of Gli family transcription factor Gli2 [8]. Gli2 translocates into the nucleus and induces the transcription of SHH target genes, such as Gli1, a reliable marker of SHH signaling [8], [9]. Gli2 is a critical component of SHH signaling and its inactivation leads to an inhibition of SHH signaling. These Gli transcription factors turn on genes in the nucleus that promote cellular proliferation, cellular survival, stemness, and cell fate determination in a variety of organs [5], [10]. SHH pathway is a morphogen required for proper pattern formation during embryogenesis; however, deregulation of this pathway is responsible for several human cancers [8], [10], [11]. Recent evidence indicates that SHH signaling pathway at the level of Gli genes has a critical role in normal pancreas development and there is mounting evidence that dysregulated SHH signaling plays some role in pancreatic cancer [12]. Furthermore, several reports indicate that human pancreatic cancers over express Gli genes [13], [14].

Transcription factors of the Gli family have dual functions such as activator and repressor that are defined only partially and can respond to combinatorial and cooperative Gli activity. The Gli family plays critical roles in the mediation and interpretation of SHH signals [15]. SHH-driven cancers arise from a variety of mutations that affect different components, including the key transcriptional effector Gli proteins, leads to a variety of human malignancies including medulloblastoma, rhabdomyosarcoma, melanoma, basal cell carcinoma, and breast, lung, liver, stomach, prostate, and pancreatic cancers [16], [17], [18], [19], [20]. Constitutively, SHH-Gli signaling is active in basal cell carcinomas, medulloblastomas and cancers of esophagus, due to mutation in Patched or Smoothened [21], [22]. Melanomas and carcinomas of the prostate have further shown a SHH-Gli signaling axis [23]. In gastrointestinal cancers, SHH signaling activation occurs through transcriptional up regulation of the SHH ligand [24]. It has recently been suggested that SHH signaling progresses during colon carcinogenesis [25], [26] and in metastatic disease [27] whereas in normal colonic tissue, SHH signaling is involved in differentiation [28]. Recently, genes have been profiled that are regulated downstream of Gli1 and Gli2 that are involved in cell proliferation and cell cycle [29], [30], and cell survival (PDGFRα and Bcl-2) [22]. Gli2 is also expressed in many basal cell carcinomas [31], suggesting that these genes might also be involved in the development of PC, which could be consistent with its partial action as mediator of SHH signals [32]. However, the roles of Gli genes (Gli1 and Gli2) in SHH-driven cellular survival and cell death responses remain ill-defined, and specifically, their role in cellular proliferation and survival of pancreatic CSCs is unknown and the downstream target genes involved in determination of cell fate.

Much attention has been recently focused on the role of cancer stem cells (CSCs)/cancer initiating cells (CICs) in the initiation and progression of solid malignancies. CSCs may be responsible for tumor onset, self-renewal/maintenance, mutation accumulation, and metastasis due to their ability to express anti-apoptotic and drug resistant proteins, thus sustaining tumor growth [33], [34]. The SHH signaling pathway is a key regulator of physiological cell processes which include proliferation, differentiation, and apoptosis [35]. Recent studies indicate that SHH signaling system plays a key role also in CSC biology including in the regulation of CSCs self-renewal, differentiation; and tumorigenic potential, suggesting SHH signaling could be a promising therapeutic target in PCs [14]. Activating SHH signaling may abrogate the resistance of CSCs to chemotherapy and could lead to the development of novel therapeutic approaches for the treatment of PCs.

To identify downstream targets of the Gli genes that regulate cellular proliferation and survival in pancreatic cancer stem cells (CSCs), we employed an inhibitor of SHH signaling, GDC-0449 (Smoothened inhibitor), which has been identified in a cell-based small molecule screen for inhibitors of Gli family-mediated transcription [36]. GDC-0449 acts as a potent inhibitor of Smoothened and shows a high degree of selectivity for SHH-Gli signaling [36]. In human pancreatic CSCs, we showed the inhibition of the SHH signaling pathway by targeting the Gli transcription factors. GDC-0449 induced significant cell death in three pancreatic cancer cell lines (AsPC-1, PANC-1 and MIA PaCa-2) and pancreatic CSCs. In further detailed analyses of pancreatic CSCs, GDC-0449 decreased SHH signaling components (Gli1, Gli2, Patched-1, Patched-2, SHH and Smoothened) expression, Gli-DNA binding and Gli-luciferase reporter activities. In addition, knockdown of both Gli1 and Gli2 expressions using shRNA conferred resistance to GDC-0499-induced cytotoxicity. Furthermore, GDC-0449 decreased the expression of PDGFRα concomitant with elevated levels of Fas, increased the expression of TRAIL-R1/DR4 and TRAIL-R2/DR5, decreased Bcl-2 expression, and induced caspase-3 activity and PARP cleavage. GDC-0449-induced changes in gene expression and apoptosis were blocked by Gli1 plus Gli2 shRNA, thus pointing a role of Gli for cellular proliferation and survival in human pancreatic CSCs. These data suggest that GDC-0449 suppresses pancreatic CSCs proliferation and survival by inhibiting SHH signaling pathway at the level of Gli genes.

## Results

### SHH pathway signaling components are expressed in human pancreatic cancer cell lines and pancreatic CSCs

**Figure 1:**
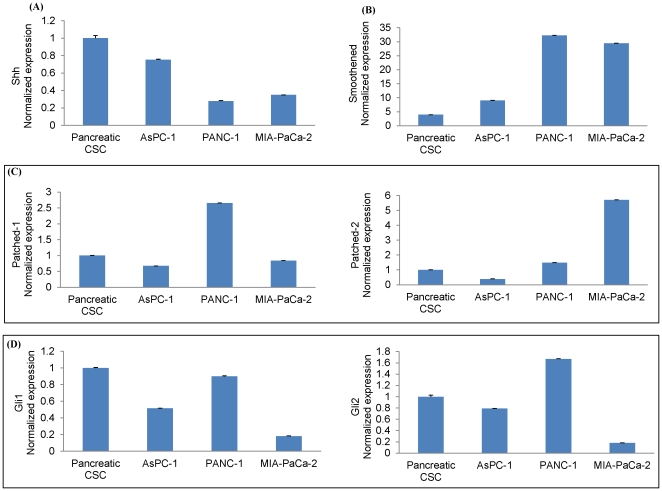
Expressing components of Sonic Hedgehog (SHH) signaling pathway in human pancreatic cancer cell lines and pancreatic cancer stem cells (CSCs). Pancreatic cancer cells (AsPC-1, PANC-1 and MIA PaCa-2) and pancreatic CSCs were grown for 48 h. Total RNA was isolated and expression of Shh, Patched-1, Patched-2, Smoothened, Gli-1 and Gli-2 was measured by qRT-PCR. HK-GAPD was used as endogenous normalization control. All assays were performed in triplicate and were calculated on the basis of ΔΔ*C*t method. The n-fold change in mRNAs expression was determined according to the method of 2^-ΔΔCT^ with GAPDH employed as the endogenous control.

We first measured the expression of various components of SHH pathway in human pancreatic cancer AsPC-1, PANC-1, and MIA-PaCa-2 cell lines and pancreatic CSCs by RT-PCR (Fig. 1). The data demonstrate that the components of SHH signaling pathway, including the ligand (Shh), the signaling molecules (Patched-1, Patched-2 and Smoothened) and effectors (Gli1, and Gli2) are expressed in human pancreatic cancer cell lines and pancreatic CSCs. These data suggest that SHH pathway is intact in pancreatic cancer cell lines and CSCs, and supports the concept that the binding of the Shh ligand to the Patched receptor diminishes its inhibitory effects on Smoothened, allowing signal transduction that will result in activation and nuclear translocation of Gli family transcription factors [13], [37].

### GDC-0449 reduces cell viability and induces apoptosis in human pancreatic cancer cell lines and pancreatic CSCs

**Figure 2:**
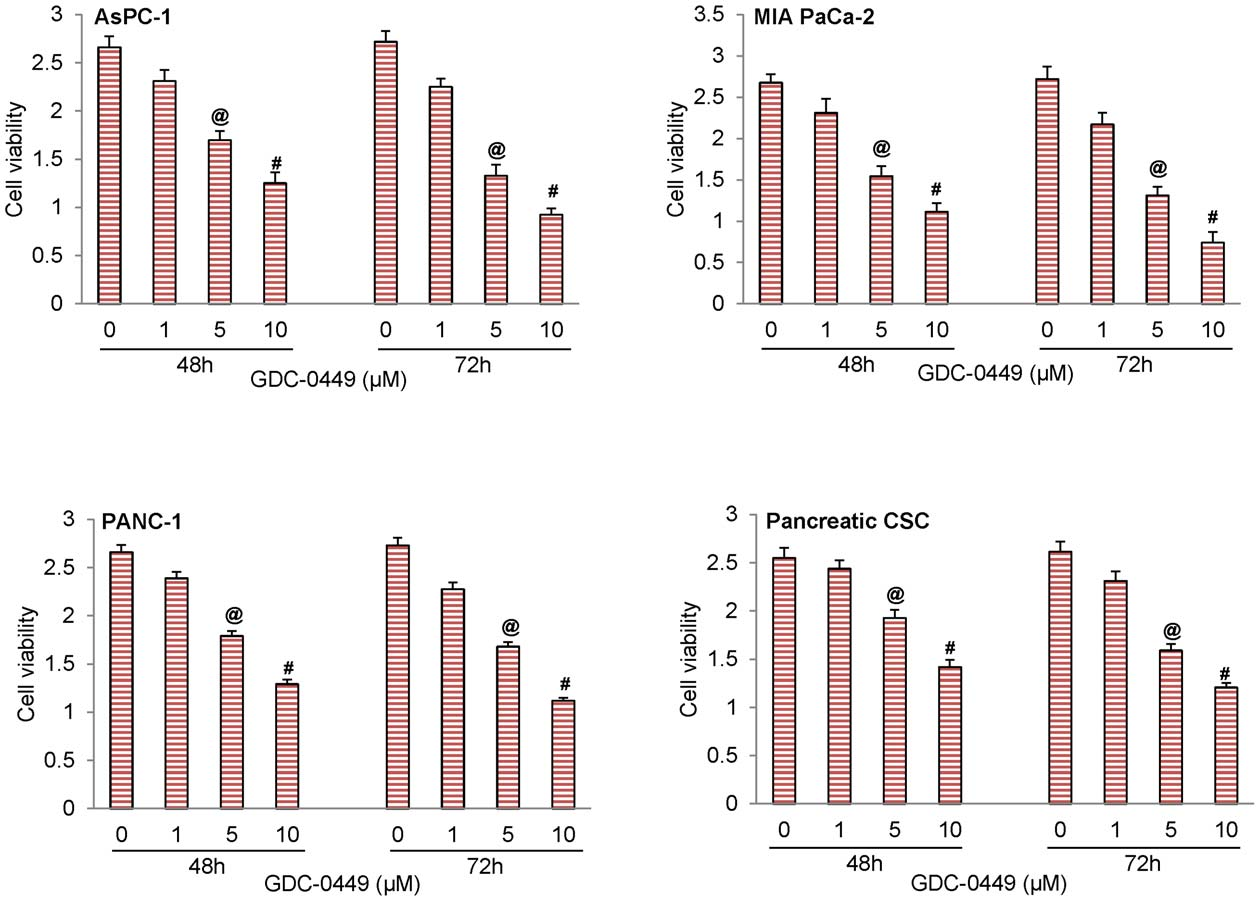
Inhibition of SHH signaling suppressed cell viability in human pancreatic cancer cell lines and pancreatic CSCs by GDC-0449. Cells were treated with GDC-0449 (0, 1, 5 and 10 µM) for 48 and 72 h. At the end of incubation period, cell viability was measured by XTT in (A) AsPC-1, (B) MIA PaCa-2, (C) PANC-1, and (D) Pancreatic CSCs. Data represent mean ± SD. @ or # significantly different from respective control (P<0.05).

**Figure 3:**
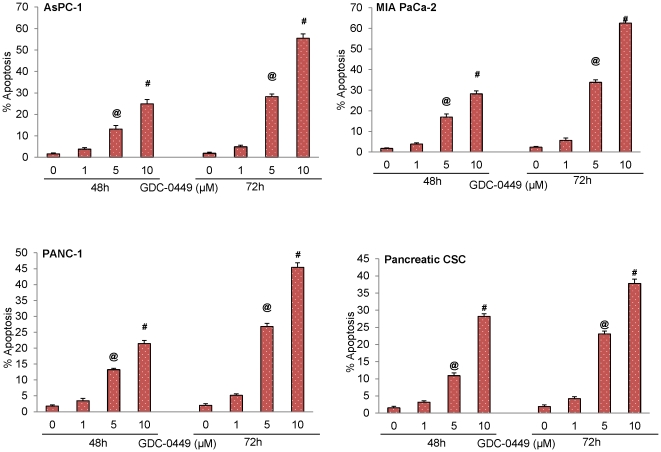
Induction of apoptosis by GDC-0449 in pancreatic cancer cell lines and pancreatic CSCs. The cells were treated with GDC-0449 (0, 1, 5 and 10 µM) for 48 and 72 h. At the end of incubation period, cells were harvested and apoptosis was measured. Data represent mean ± SD. @ or # significantly different from respective control (P<0.05).

Previous clinical studies have suggested that GDC-0449 is a small molecule inhibitor of Smoothened. We first sought to examine the effects of GDC-0499 on cell viability and apoptosis in the panel of three human pancreatic cancer cell lines and pancreatic CSCs. Inhibition of cell survival and induction of apoptosis was observed within 24 h following exposure to this drug (data not shown), but was maximally noticed at 72 h (Figs. 2 and 3). In all the cell lines, GDC-0449 induced apoptosis is a dose-dependent manner reaching up to 65%. By comparison, GDC-0449 was less effective in inducing apoptosis in CSCs (Fig. 3). For further mechanistic studies we have used pancreatic CSCs.

### GDC-0449 regulates downstream targets of SHH pathway, inhibits Gli-DNA interaction, Gli transcriptional activity and decreases Gli nuclear translocation in pancreatic CSCs

**Figure 4:**
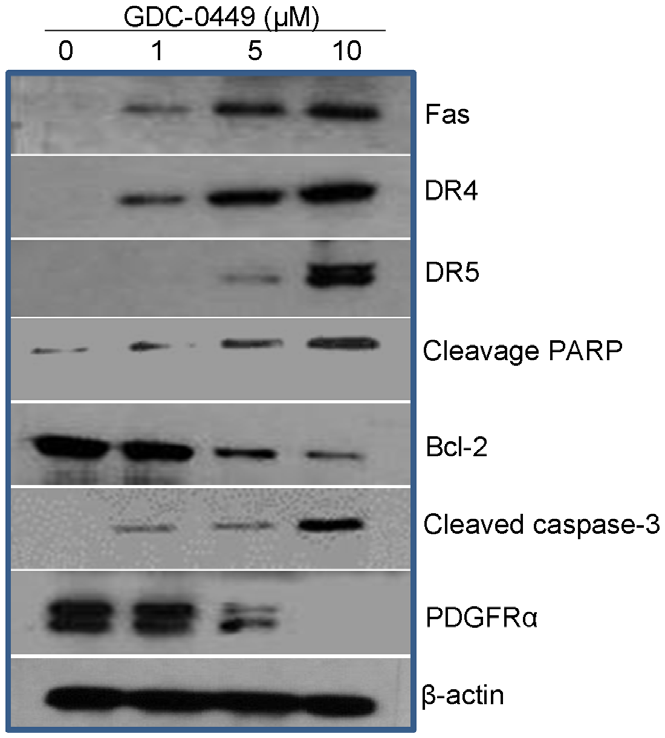
GDC-0449 differentially regulates genes involved in the balance between cell death and cell survival. Pancreatic CSCs were treated with GDC-0449 (0, 1, 5 and 10 µM) for 48 h. The expression of Fas, DR4/TRAIL-R1, DR5/TRAIL-R2, PARP cleavage, Bcl-2, and Caspase-3 by the Western blot analysis. β-Actin was used as a loading control.

We next examined the effect of GDC-0449 on the expression of Fas, DR4/TRAIL-R1, DR5/TRAIL-R2, PARP, Bcl-2, caspase-3, and PDGFRα by the western blot analysis (Fig. 4). GDC-0449 induced the expression of Fas, DR4, and DR5 and inhibited the expression of Bcl-2 and PDGFRα in pancreatic CSCs. In addition, GDC-0449 induced the cleavage of caspase-3 and PARP in pancreatic CSCs, correlating with the extent of cell survival and apoptosis.

**Figure 5:**
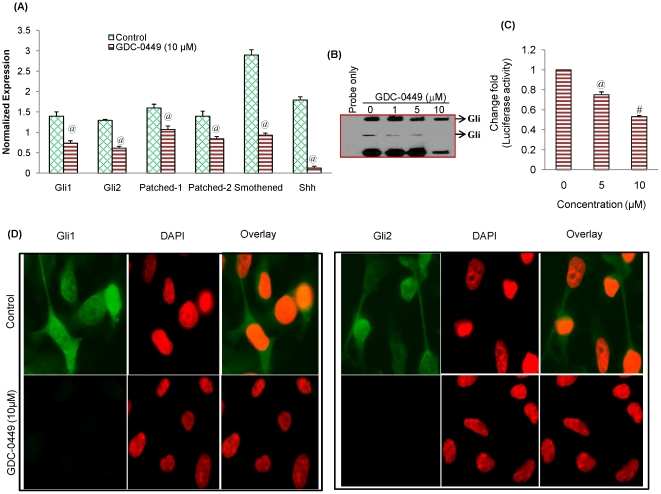
GDC-0449 downregulates the expression of various components of SHH signaling pathway in pancreatic CSCs . (A), Pancreatic CSCs were treated with GDC-0449 (10 µM) for 36 h. At the end of incubation period, RNA was extracted and the expression of Gli1, Gli2, Patched-1, Patched-2, Smoothened and Shh was measured by qRT-PCR. Data represent the mean ± SD. @ significantly different from respective control (P<0.05). (B), Pancreatic CSCs were treated with and GDC-0449 (0, 1, 5 and 10 µM) for 48 h. Nuclear extracts were prepared and the gelshift experiment was performed. Probe only, control (without GDC-0449), and GDC-0449 treated samples (1, 5 and 10 µM), respectively. The data are representative of three experiments. (C), Gli-dependent luciferase activity is reduced by GDC-0499. Pancreatic CSCs were transduced with lentiviral construct expressing Gli-dependent luciferase reporter, and treated with GDC-0449 (0, 5 and 10 µM) for 48 h. Lysates were prepared, and luciferase activity was measured. Normalized luciferase activity is presented as mean ± SD. @ or # significantly different from respective control (P< 0.05). (D), GDC-0449 inhibits expression of Gli1 and Gli2 in human pancreatic CSCs. The cells were seeded on fibronectin-coated coverslips and treated with GDC-0449 (10 µM) for 48 h. Subsequently, cells were fixed with 4% paraformaldehyde, blocked in 10% normal goat serum and stained with Gli1 and Gli2 primary antibodies (1∶100) for 16 h at 4°C and washed with PBS. Afterwards, cells were incubated with fluorescently labeled secondary antibody (1∶200) along with DAPI (1 mg/ml) for 1 h at room temperature and cells were mounted and visualized under a fluorescent microscope. For better visuality, the color of DAPI was changed from blue to red.

We next sought to examine the effects of GDC-0449 on SHH pathway by measuring the expression of SHH receptors (Patched-1, Patched-2 and Smoothened) and effectors (Gli1 and Gli2) by qRT-PCR (Fig. 5A). GDC-0449 inhibited the expression of smoothened, Patched-1, and Patched-2. Similarly, GDC-0449 inhibited the expression of transcription factor Gli1 and Gli2. These data suggest that GDC-0449 can regulate CSC characteristics by inhibiting various components of SHH pathway.

Since Gli mediates the effects of Shh, we next examined the Gli-DNA interaction by electrophoretic mobility shift assay (EMSA) in human pancreatic CSCs. Treatment of CSCs with GDC-0449 resulted in decreased Gli-DNA binding activity in a dose-dependent manner (Fig. 5B).

We next examined the effect of GDC-0449 on Gli transcriptional activity (Fig. 5C). Exposure of CSCs with GDC-0449 for 36 h resulted in inhibition of Gli-dependent luciferase reporter activity in a dose-dependent manner. GDC-0449 inhibited the expression of Patched-1 and Patched-2 because they are downstream targets of Gli. We next employed immunofluorescence technique to examine the effects of GDC-0449 on nuclear expression of Gli (Fig. 5D). Pancreatic CSCs were treated with GDC-0449, and the nuclear expression of Gli1 and Gli2 was observed. GDC-0449 inhibited the nuclear expression of Gli1 and Gli2. Overall, these data suggest that GDC-0449 can inhibit Gli DNA binding and transcriptional activity.

### Human pancreatic CSCs require active Gli function for sustained expression of genes involved in the cell survival and proliferation

**Figure 6:**
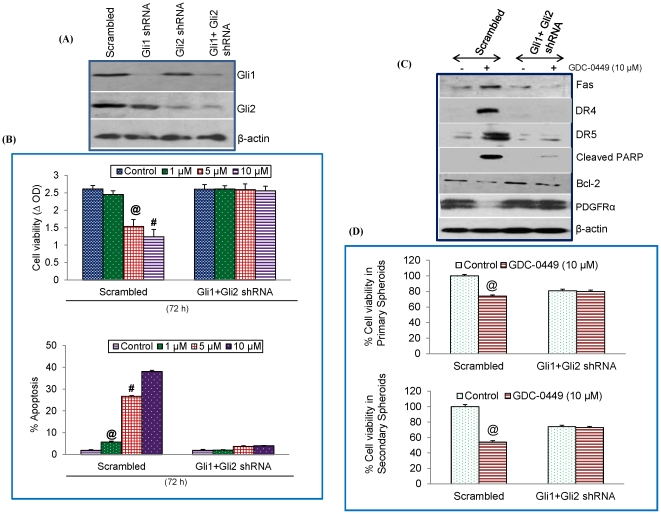
Impact of SHH signaling pathway on the regulation of cell survival and antiproliferative effects of GDC-0449. (A), Knockout of Gli1 shRNA and Gli2 shRNA in human pancreatic CSCs. Pancreatic CSCs were transduced with lentiviral particles expressing Scrambled, Gli1 shRNA, Gli2 shRNA or Gli1 plus Gli2 shRNA (KO). (B), Pancreatic CSCs were treated with GDC-0449 (0, 1, 5 and 10 µM) for 72 h, and cell viability and apoptosis was measured in scrambled and Gli1 plus Gli2 shRNA CSCs. Data represent mean ± SD, n = 4. @ or # significantly different from respective control (P<0.05). (C), Scrambled and Gli1 plus Gli2 shRNA pancreatic CSCs were treated with GDC-0449 (0 and 10 µM) for 48 h, and lysates were extracted to determine the expression of DR4, DR5, PDGFRα, Fas and Bcl-2 by Western blot analysis. β-Actin was used as the loading control. (D), Inhibition of primary and secondary spheroids by GDC-0449. Pancreatic CSCs (scrambled, and Gli1 + Gli2 shRNA) were seeded in suspension and treated with GDC-0449 (10 µM) for 7 days. At the end of incubation period, spheroids were collected, and dissociated with Accutase (Innovative Cell Technologies, Inc.). For secondary spheroids, cells were reseeded and treated with GDC-0449 (10 µM) for additional 7 days. Cell viability was measured by trypan blue assay. Data represent mean ± SD. @ or # significantly different from respective controls, P<0.05.

In order to examine the effects of Gli1 and Gli2 on cell proliferation, apoptosis and down-stream targets, we inhibited the expression of Gli1 and Gli2 transcription factors by shRNA. As shown in Fig. 6A, lentiviral mediated expression of Gli1 and Gli2 shRNA inhibited the expression of Gli1 and Gli2 proteins in pancreatic CSCs. If GDC-0449 inhibits cell viability and induces apoptosis by inhibiting Gli transcription factors, the inhibition of Gli1 and Gli2 should block the anti-proliferative and pro-apoptotic effects of GDC-0449. To confirm the Gli-dependent anti-proliferative and pro-apoptotic effects GDC-0449, we used pancreatic CSCs expressing Gli1 + Gli2 shRNA (Fig. 6B). GDC-0449 inhibited cell viability and induced apoptosis in CSCs/scrambled cells. Inhibition of Gli1 and Gli2 alone by shRNA was unable to completely inhibit the effects of GDC-0449 on CSC's viability (data not shown). By comparison, inhibition of Gli1 plus Gli2 together by shRNA suppressed the effects of GDC-0449 on cell viability and apoptosis in CSCs. These data suggest that both Gli1 and Gli2 genes are required for anti-proliferative and pro-apoptotic effects of GDC-0449.

We next examined the effects of inhibiting Gli expression on downstream target of SHH pathway by Western blot analysis (Fig. 6C). GDC-0449 (10 µM) induced the expression of Fas, DR4 and DR5, cleaved PARP, and inhibited the expression of Bcl-2 and PDGFRα in CSC/scrambled cells. It is interesting to note that the expression of PDGFRα was decreased following GDC-0449 treatment with concomitant increase in Fas. By comparison, GDC-0449 had no significant effects on expression of these downstream targets of SHH signaling pathway in CSCs/Gli1 + Gli2 shRNA cells.

We next examined the effect of GDC-0449 on primary and secondary spheroid formation (Fig. 6D). GDC-0449 inhibited the formation of primary and secondary spheroids in CSC/scrambled cells. By comparison, transduction of Gli1 plus Gli2 shRNA in CSCs suppressed the inhibitory effects of GDC-0449 on primary and secondary spheroid formation. These data suggest that GDC-0449 can inhibit pancreatic CSC characteristics.

## Discussion

Our study demonstrates, for the first time, that Smoothened-dependent therapeutic agent GDC-0449 regulates cellular survival in human pancreatic CSCs. Specifically, GDC-0449 inhibited cell viability and induced apoptosis in three pancreatic cancer cell lines and pancreatic CSCs. In pancreatic CSCs, GDC-0449 also suppressed the cell viability, Gli-DNA binding and Gli-transcriptional activities, induced apoptosis through caspase-3 activation and PARP cleavage. Analysis of the molecular mechanisms of GDC-0449-induced cell death in CSCs showed increased Fas expression and decreased expression of PDGFRα, which also regulates Fas. Furthermore, Bcl-2 was down-regulated whereas DR4 and DR5 expressions were increased following the treatment of GDC-0449. Suppression of both Gli1 plus Gli2 by shRNA mimicked the changes in cell viability, spheroid formation, apoptosis and death-related gene expression observed in GDC-0449-treated pancreatic CSCs. Thus, activated Gli genes repress DRs and Fas expressions while up-regulating Bcl-2 and PDGFRα expressions to inhibit Fas. Our results highlight the critical role of SHH signaling in human pancreatic CSCs and the possibility of targeting Gli1 and Gli2 activator functions using GDC-0449 in pancreatic cancer stem cells.

SHH signaling events have been implicated in tumor cell proliferation and survival as well as is the molecular hallmark of different human tumor entities that include esophageal squamous cell carcinoma, basal cell carcinoma [38], subsets of medulloblastoma [39], prostate cancer [40], colon cancer [41], brain tumors [42], rhabdomyosarcoma [43], and breast cancer [44]. Multiple lines of evidence support the idea that SHH signaling is a prerequisite for the increased viability of pancreatic CSCs, such that blocking active SHH signaling with the therapeutic inhibitor molecules such as GANT-61 (targeting effectors Gli1 and Gli2) and cyclopamine (targeting SHH receptor Smoothened) induced cell death [45]. We have shown that human pancreatic cancer cell lines and CSCs consistently express various components of SHH pathway including effectors (Gli1 and Gli2), and receptors (Patched-1, Patched-2, and Smoothened), and most importantly the ligand: SHH, suggesting, SHH pathway is one of the “core” signaling pathway or an autocrine mode of SHH signaling in these cells. Activation of the SHH signaling cascade consistently induces Gli family transcription factors (Gli1 and Gli2), hence both Gli genes mRNA [46], expressed in pancreatic cancer cell lines and CSCs, stating potential involvement of SHH signaling in human pancreatic carcinogenesis.

Aberrant activation of SHH signaling is implicated in many human cancers. To identify new therapeutic targets, inhibition of SHH signaling has been attempted in multiple human cancers [11], [41], [45]. Recently, Gli and Smo antagonists have been used to abrogate SHH signaling in human cancers (40). Natural and synthetic Smoothened antagonists such as cyclopamine [24] and IPI-926 [11], respectively have inhibited proliferation, survival and have anti-tumor functions, resulting in abrogation of SHH signaling. However, during the clinical trials various levels of resistance have been observed. Pharmacologic agents including cyclopamine (11), GDC-0499 and GANT-61 [41] have inhibited survival and antitumor functions in preclinical models of human cancers. Gli genes are potential members of SHH pathway, which act as downstream mediators of SHH signaling and they have regulatory effects on cell cycle and apoptosis. Hence, one potential druggable target lies in Gli family transcription factors, which are the final mediators of transcriptional regulation in the SHH signaling pathway. In the present study, exposure to GDC-0449 induces significant cytotoxicity and apoptosis in human pancreatic cancer cells and CSCs.

**Figure 7:**
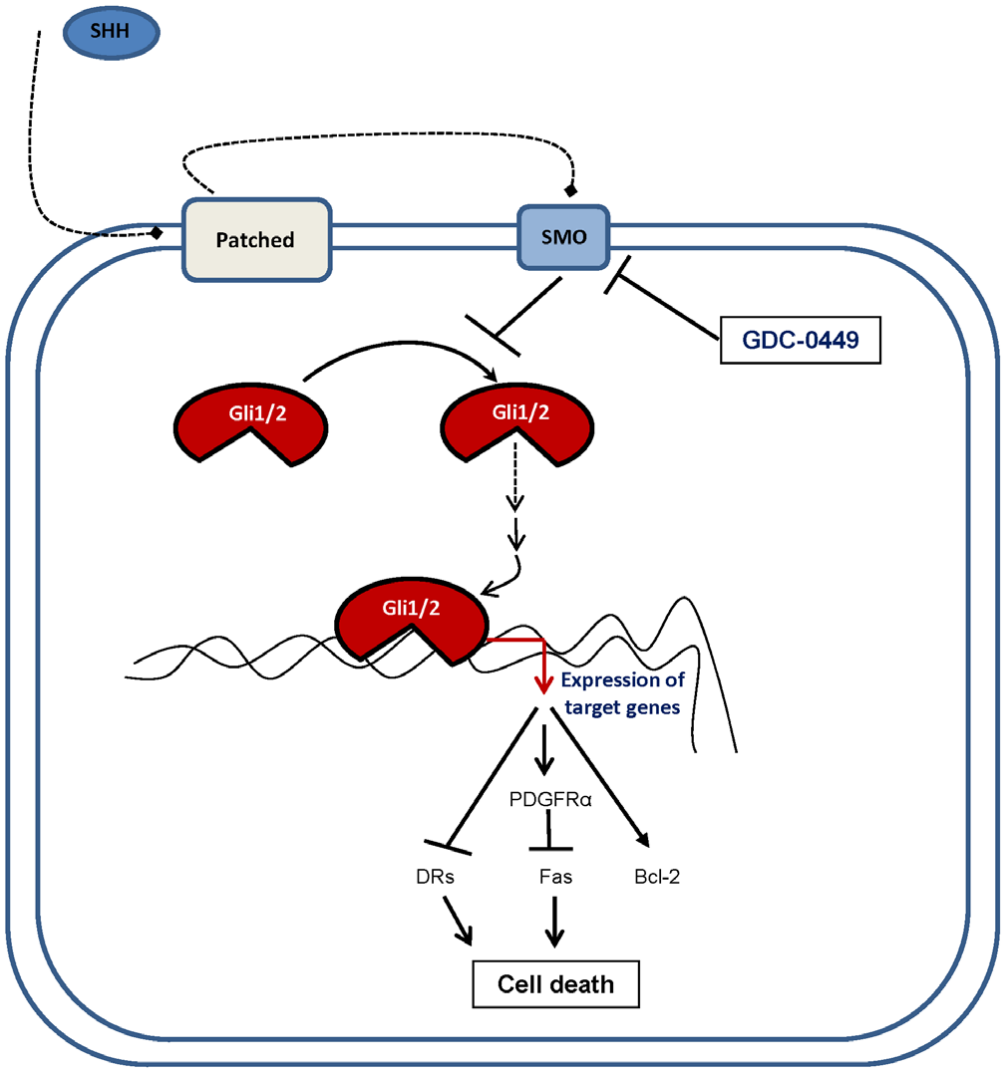
Schematic representation of the inhibition of SHH signaling and genes involved in the balance between cellular proliferation and cell death. Activated Gli1 and Gli2, downstream of SHH-Patched-Smoothened, regulate targets of SHH signaling including Bcl-2, PDGFRα, Fas, and DRs. GDC-0449 (targeting Smoothened) blocks the indirect functions of Gli activators, resulting in cell death.

GDC-0449 treatment effectively decreased Gli-DNA binding and Gli-transcriptional activity in human pancreatic CSCs. These results are suggesting that GDC-0449 may induce posttranscriptional alterations of Gli gene, which might point towards, an increase in phosphorylation that either prevent DNA binding or destabilize the Gli-DNA complex. Post-translational modifications of Gli gene is an important mechanism that regulates the ability of different transcription factors to inhibit distinct gene sets, involved in regulation of cell death inhibition [41], consistent with previous observations in pancreatic cancer cells engineered to express Gli1 [13]. Of particular interest, Smoothened-dependent GDC-0449 treatment of pancreatic CSCs markedly inhibited expressions of SHH receptors (Patched-1, Patched-2 and Smoothened) and effectors (Gli1 and Gli2), thereby showing potential for therapeutic application for treatment of pancreatic cancer. It was previously reported that GDC-0449 was identified as inhibitor of Gli1 transcriptional activity, and also abrogated Gli2-mediated transcription [36]. Subsequently, we have observed that reduction in Gli2 expression preceded that of Gli1 in pancreatic CSCs. Further, studies in mice have revealed that Gli2 is primary mediators of SHH signaling and is known to transcriptionally regulate Gli1 expression [25]. Because the SHH signaling pathway is already activated in human pancreatic CSCs, studies using shRNA knockdown of Gli1 and Gli2 alone and simultaneously were conducted. GDC-0449 significantly inhibited cell viability and induced apoptosis in shRNA Gli1 and Gli2 alone (data not shown). Interestingly, significant protection from GDC-0449-induced cytotoxicity and apoptosis was recorded in shRNA knockdown of both Gli1 and Gli2. These data further support the idea that GDC-0449 may have Gli-dependent and -independent mode of action (Fig. 7) and further show the importance of functional Gli genes in maintaining cellular survival in human pancreatic CSCs.

To assess antiproliferative effect of GDC-0449 in more detail, we next correlated the expression of cell cycle and apoptosis-related genes with SHH signaling in pancreatic CSCs. In several stem cells and cancer cells, SHH acts as a survival factor [9], [47]. SHH has been reported to control the proliferation of several cell types through various molecular mechanisms such as increase in PDGFRα and Bcl-2 expressions, decrease in DRs and Fas expressions, and inhibition of PARP cleavage and caspase-3 activation in colon cancer cell lines overexpressing Gli genes [41] or, an overexpression of dominant-negative FADD in cells expressing SHH or a constitutively active Smoothened [41]. In our study, GDC-0449 induced a marked increase in the expression of Fas and both DRs (TRAIL-R1/DR4 and TRAIL-R1/DR5), suggesting the potential involvement of these DRs in drug-induced apoptosis. Recent reports indicated the absence of Gli binding sites in the promoter region of DR4 and DR5. The regulation of DR expression by the Gli is currently unknown and may be via an indirect mechanism. However, GDC-0449 induced up regulation of both DRs in pancreatic CSCs, suggesting transcriptional regulation of DRs by a currently unknown mechanism. We also determined the contributions of apoptotic cell death pathways (mitochondria-mediated intrinsic and death receptor signaling-mediated extrinsic), based on the known regulation of PDGFRα upstream of Fas [2], [48], and of Bcl-2, which may be a direct transcriptional target of both Gli1 and Gli2, a key anti-apoptotic protein in SHH-dependent cell survival [49]. We have shown that GDC-0449 treatment reduces pro-survival protein Bcl-2 expression in pancreatic CSCs. Thus, our data demonstrate that activation of SHH pathway can regulate genes involved in both cell-extrinsic and cell-intrinsic pathways of apoptosis.

Together, this study reveals that endogenous SHH signaling controls the self-renewal capacity of human pancreatic CSCs by targeting down-stream targets of Gli. In conclusion, GDC-0449 can be used for the treatment of human pancreatic cancer because it can target pancreatic cancer CSCs.

## Methods

### Antibodies and reagents

Antibodies against SHH, Smoothened, Fas, TRAIL-R1/DR4, TRAIL-R2/DR5, and β-actin were purchased from Santa Cruz Biotechnology Inc. (Santa Cruz, CA). Antibodies against Gli1, Gli2, Patched-1, Patched-2, PDGFRα and caspase-3 were obtained from Cell Signaling Technology (Danvers, MA). Enhanced chemiluminescence (ECL) Western blot detection reagents were from Amersham Life Sciences Inc. (Arlington Heights, IL). GDC-0449 (Vismodegib; C19H14Cl2N2O3S) was purchased from Tocris (Ellisville, MO). All other chemicals used were of analytical grade and were purchased from Fisher Scientific (Suwanee, GA) and Sigma-Aldrich (St. Louis, MO).

### Cell culture

Pancreatic cancer cell lines (AsPC-1, PANC-1 and MIA PaCa-2) were obtained from American Type Culture Collection (Manassas, VA) and cultured in RPMI 1640 supplemented with 10% fetal bovine serum (FBS) and 1% antibiotic-antimycotic at 37°C in a humidified atmosphere of 95% air and 5% CO_2_. Human pancreatic CSCs (CD133^+^/CD44^+^/CD24^+^/ESA^+^) were obtained from Celprogen Inc. (San Pedro, CA). They were isolated from primary tumors and have been described previously [33]. The CSCs were cultured in DMEM supplemented with 1% N2 Supplement (Invitrogen), 2% B27 Supplement (Invitrogen), 20 ng/ml human platelet growth factor (Sigma-Aldrich), 100 ng/ml epidermal growth factor (Invitrogen) and 1% antibiotic-antimycotic (Invitrogen) at 37°C in a humidified atmosphere of 95% air and 5% CO_2_. Pancreatic CSCs were routinely verified by morphology and growth characteristics.

### Lentiviral particle production and Gli1 shRNA and Gli2 shRNA transduction

Gli1 shRNA (5′-GCCTGAATCTGTGTATGAA-3′; 5′- GTTTGAATCTGAATGCTAT-3′; 5′- AGCTAGAGTCCAGAGGTTC-3′; 5′- CCGGAGTGCAGTCAAGTTG-3′ and 5′- GGCTGGACCAGCTACATCA-3′) and Gli2 shRNA (5′- CCGAGAAGCAAGAAGCCAA-3′; 5′- CACAGCATGCTCTACTACT-3′; 5′- TCGCTAGTGGCCTACATCA-3′; 5′-TCCGAGAAGCAAGAAGCCA-3′ and 5′- CCAGACGACGTGGTGCAGT-3′) were obtained from Open Biosystems, Huntsville, AL) and cloned into TRIPZ vector. Lentiviral particles were produced by triple transfection of HEK 293T cells. Packaging 293T cells were plated in 10-cm plates at a cell density of 5×10^6^ a day prior to transfection in DMEM containing 10% heat-inactivated fetal bovine serum without antibiotics. Transfection of packaging cells and infection of pancreatic CSCs were carried out using standard protocols [50] with some modifications. In brief, 293T cells were transfected with 4 µg of plasmid and 4 µg of lentiviral vector using lipid transfection (Lipofectamine/Plus reagent, Invitrogen) according to the manufacturer's protocol. Viral supernatants were collected and concentrated by adding PEG-it virus precipitation solution (SBI System Biosciences) to produce virus stocks with titers of 1×10^8^ to 1×10^9^ infectious units/ml. Viral supernatant was collected for three days by ultracentrifugation and concentrated 100-fold. Titers were determined on HEK293T cells. Pancreatic CSCs were transduced with lentivirus expressing scrambled shRNA (control), Gli1 shRNA, or Gli2 shRNA. Pancreatic CSCs simultaneously expressing both Gli1 plus Gli2 shRNA were also generated. Following transduction, the CSCs were washed 3 times with Difco's 1X PBS and allowed to grow for 3 passages before screening for gene expression. Once decreased expression of the targeted gene was confirmed, the cells were used for experiments. Stable expression of Gli1 ShRNA or Gli2 shRNA was ensured by culturing cells in the presence of a selection antibiotic puromycin (5.0 µg/ml), whereas induction of both Gli1 shRNA and Gli2 shRNA was performed in the presence of doxycycline (2.0 µg/ml). The transduced CSCs were washed three times with PBS (without Ca^++^ or Mg^++^) and used for experiments.

### Cell viability and apoptosis assays

Cells (1.5 × 10^4^) were incubated with 0-10 µM of GDC-0449 in 250 µl of culture medium in 96-well plate for 48 and 72 h before cell viability determination. Cell viability was determined by the XTT assay. In brief, a freshly prepared XTT-PMS labeling mixture (50 µl) was added to the cell culture. The absorbance was measured at 450 nm with λ correction at 650 nm. The cell viability was expressed as ΔOD (OD_450_ - OD_650_). The apoptosis was determined by FACS analysis of propidium iodide (PI)-stained cells. In brief, cells were trypsinized, washed with PBS and resuspended in 200 µl PBS with 10 µl RNAase (10 mg ml/ml) and incubated at 37°C for 30 min. After incubation, 50 µl PI solution was added and cells were analyzed for apoptosis using a flow cytometry (FACSCalibur, BD Biosciences, San Jose, CA).

### Tumor spheroid assay

For spheroid forming assay, cells were plated in six-well ultralow attachment plates (Corning Inc., Corning, NY) at a density of 1,000 cells/ml in DMEM supplemented with 1% N2 Supplement (Invitrogen), 2% B27 Supplement (Invitrogen), 20 ng/ml human platelet growth factor (Sigma-Aldrich), 100 ng/ml epidermal growth factor (Invitrogen) and 1% antibiotic-antimycotic (Invitrogen) and incubated at 37°C in a humidified atmosphere of 95% air and 5% CO_2_. Cells were treated with GDC-0449 (10 µM) and spheroids were collected by dissociation with Accutase (Innovative Cell Technologies, Inc.) after 7 days. The CSCs obtained from dissociation were counted by coulter counter using trypan blue dye.

### Western blot analysis

Whole cell lysates were extracted from GDC-0449-treated cells using RIPA lysis buffer containing 1 X protease inhibitor cocktail. Protein concentrations were determined using the Bio-Rad Protein Assay (Bio-Rad, Hercules, CA). Cell lysates containing 50 µg of protein were loaded and separated on 10% Tris-HCl gel. Proteins from the gel were transferred on polyvinylidene difluoride membranes and subsequently blocked in blocking buffer [5% nonfat dry milk in 1 X Tris Buffer Saline (TBS)] and incubated overnight with primary antibodies. Membranes were washed three times with TBS-T for 10, 5, and 5 min each. After washing, membranes were incubated with secondary antibodies conjugated with horseradish peroxidase at 1∶5,000 dilution in TBS for 1 h at room temperature. Membranes were again washed three times in TBS-T and developed using ECL Substrate. Protein bands were visualized on X-ray film using an enhanced chemiluminescence system.

### RNA isolation and mRNA expression analysis

Total RNA was isolated using the Qiagen RNeasy Mini Kit according to the manufacturer's protocol (Qiagen, Valencia, CA). Briefly, RNA was isolated and reverse transcribed. cDNA reactions were amplified with QPCR SYBR Green Mix. Primers specific for each of the signaling molecules were designed using NCBI/Primer-BLAST and used to generate the PCR products. For the quantification of gene amplification, Real-time PCR was performed using an ABI 7300 Sequence Detection System in the presence of SYBR- Green. The following gene-specific primers were used:

Smoothened (5′-TCG CTA CCC TGC TGT TAT TC -3′, 5′-GAC GCA GGA CAG AGT CTC AT-3′)

Patched1 (5′-TGA CCT AGT CAG GCT GGA AG-3′, 5′-GAA GGA GAT TAT CCC CCT GA-3′)

Patched2 (5′-AGG AGC TGC ATT ACA CCA AG-3′, 5′-CCC AGG ACT TCC CAT AGA GT-3′)

Gli1 (5′-CTG GAT CGG ATA GGT GGT CT -3′, 5′- CAG AGG TTG GGA GGT AAG GA -3′)

Gli2 (5′-GCC CTT CCT GAA AAG AAG AC -3′, 5′- CAT TGG AGA AAC AGG ATT GG -3′)

HK-GAPD (5′-GAG TCA ACG GAT TTG GTC GT-3′, 5′-TTG ATT TTG GAG GGA TCT CG-3′)

Target sequences were amplified at 95°C for 10 min, followed by 40 cycles of 95°C for 15 s and 60°C for 1 min. HK-GAPD was used as endogenous normalization control. All assays were performed in triplicate and were calculated on the basis of ΔΔ*C*t method. The n-fold change in mRNAs expression was determined according to the method of 2^−ΔΔCT^.

### Gli reporter assay (p-GreenFire1 Lenti-Reporter)

The cop-GFP and luciferase genes were cloned downstream of Gli-response element, containing four Gli binding motifs (pGreen Fire1-4xGli-mCMV-EF1-Neo; System Biosciences, Mountain View, CA). For *in vitro* assays, stably transduced pancreatic CSCs were plated at 5-10,000 cells per well in 12-well plates and treated with 5 and 10 µM of GDC-0449. After incubation, CSCs were analyzed for luciferase reporter activity (Promega Corp., Madison, WI).

### Immunocytochemistry

Pancreatic CSCs were grown on fibronectin-coated coverslips (Beckton Dickinson, Bedford, MA) in the absence or presence of 10 µM of GDC-0449. Subsequently, cells were fixed with 4% paraformaldehyde for 15 min, permeabilized with 0.1% Triton X-100 in 1 X PBS, washed and blocked in 10% normal goat serum. After extensive washing with PBS, cells were stained with Gli1 and Gli2 primary antibodies (1∶100) for 16 h at 4°C and washed with PBS. Afterwards, cells were incubated with fluorescien-labeled secondary antibody (1∶200) along with DAPI (1 mg ml^−1^) for 1 h at room temperature. Finally, coverslips were washed and mounted using Vectashield (Vector Laboratories, Burlington, CA). Isotype-specific negative controls were included with each staining. Stained cells were mounted and visualized under a fluorescent microscope.

### Electrophorectic mobility shift assay (EMSA)

Gli probes were end-labeled with [γ-^32^P] dATP by incubating oligodeoxyribonucleotide strands with 5 x reaction buffer and 10 U T4 polynucleotide kinase for 1 h at 37°C. Then labeled oligonucleotides were allowed to anneal at room temperature for 10 min and 20 mg protein from each sample treated with GDC-0449 (0, 1, 5 and 10 µM) was used in 25 ml binding reactions, which consisted of 1 mg poly dI-dC, in 5 X binding buffer (50 mM Tris HCl; pH 8.0, 0.75 M KCl, 2.5 mM EDTA, 0.5% Triton-X 100, 62.5% glycerol (v/v) and 1 mM DTT). To determine specificity of DNA binding, samples were incubated with or without 20 ng of unlabeled competitor DNA for 10 min at room temperature. Then 0.1 ng of labeled probe of Gli was added and samples were further incubated for 20 min at room temperature. Samples were separated on a 5% non-denaturing polyacrylamide gel in 0.5% TBE and visualized by autoradiography.

### Statistical analysis

The mean and SD were calculated for each experimental group. Differences between groups were analyzed by one or two way ANOVA using PRISM statistical analysis software (GrafPad Software, Inc., San Diego, CA). Significant differences among groups were calculated at P<0.05.
